# Facilitating Healthier Eating at Restaurants: A Multidisciplinary Scoping Review Comparing Strategies, Barriers, Motivators, and Outcomes by Restaurant Type and Initiator

**DOI:** 10.3390/ijerph18041479

**Published:** 2021-02-04

**Authors:** Melissa Fuster, Margaret A. Handley, Tamara Alam, Lee Ann Fullington, Brian Elbel, Krishnendu Ray, Terry T-K Huang

**Affiliations:** 1 Department of Health and Nutrition Science, Brooklyn College, City University of New York, Brooklyn, New York, NY 11210, USA; alamtamara530@gmail.com; 2 Department of Epidemiology and Biostatistics, School of Medicine, University of California, San Francisco, CA 94158, USA; Margaret.Handley@ucsf.edu; 3 Library, Brooklyn College, City University of New York, Brooklyn, New York, NY 11210, USA; LAFullington@Brooklyn.cuny.edu; 4 Department of Population Health, New York University Grossman School of Medicine, and Wagner Graduate School of Public Service, New York University, New York, NY 10016, USA; Brian.Elbel@nyumc.org; 5 Department of Nutrition and Food Studies, Steinhardt School of Culture, Education, and Human Development, New York University, New York, NY 10003, USA; Krishnendu.Ray@nyu.edu; 6 Department of Community Health and Social Sciences and Center for Systems and Community Design, City University of New York Graduate School of Public Health and Health Policy, New York, NY 10027, USA; Terry.Huang@sph.cuny.edu

**Keywords:** restaurant, scoping review, food environment, food retail, eating out, nutrition-related diseases

## Abstract

Restaurants are understudied yet increasingly important food environment institutions for tackling diet-related diseases. This scoping review analyzes research and gray literature (n = 171 records) to assess which healthy eating promotion strategies have been implemented in restaurants and the associated motivations, barriers, and outcomes, compared by restaurant type (corporate/chain vs. independently owned restaurants) and initiator (restaurant-initiated vs. investigator-initiated). We found that the most commonly reported strategy was the increase of generally healthy offerings and the promotion of such offerings. Changes in food availability were more common among corporate restaurants and initiated by restaurants, while environmental facilitators were more commonly initiated by investigators and associated with independently owned restaurants. Aside from those associated with revenue, motivations and barriers for healthy eating promoting strategies varied by restaurant type. While corporate restaurants were also motivated by public health criticism, independently owned restaurants were motivated by interests to improve community health. Revenue concerns were followed by food sourcing issues in corporate restaurants and lack of interest among independently owned restaurants. Among reporting sources, most outcomes were revenue positive. This study shows the need for practice-based evidence and accounting for restaurant business models to tailor interventions and policies for sustained positive changes in these establishments.

## 1. Introduction

Foods away from home have been associated with poor dietary behaviors and diet-related health outcomes [1], leading to the implementation of policies and interventions aimed at improving the consumer food environments in restaurants. These interventions have focused on improving the foods offered and facilitating healthier eating through promotion, portion control, and other environmental changes within the establishment [2,3,4,5,6,7,8,9,10,11,12]. Past review studies have focused on specific interventions, in particular, menu calorie labeling [13,14] and nudges for healthier choices, where customer behavior is intended to be altered by how choices are presented to them [15,16]. When examining consumer health and eating behavior outcomes in restaurants, these reviews have shown neither change in a reduction in calories, nor key nutrients of concerns (saturated fat, added sugar) [16], and mixed-effects from menu labeling, with some promising effects in fast food restaurants [13], or a small effect [14].

Restaurants are complex and dynamic contexts, making intervention and policy design and implementation particularly intricate. More research is needed to understand the restaurant context, and the business-focused priorities, as factors that can facilitate or hinder the successful implementation of healthy eating promotion interventions, resulting in healthier food environments. This assessment is needed, given that most of the existing review studies focus on consumer health outcomes, as briefly reviewed above. One notable exception is the review by Blake et al. (2019), examining business outcomes reporting in healthy retail strategy evaluation research studies. The review underscored the lack of emphasis on business perspectives, where reports of retailer perceptions were described as limited and mostly addressed community stewardship, or perceptions of how the business impacted health behaviors and outcomes and the level of satisfaction with the intervention [17]. Moreover, the review merges restaurants within other food retail sources, failing to fully examine restaurants as distinct contexts.

This scoping review aims to increase understanding of the restaurant context as a site for healthy eating promotion strategies. We examined consumer-facing innovation strategies restaurants have used that may promote or facilitate healthier eating. While previous reviews have focused on public health interventions described in academic journals, we have expanded our sources by incorporating media sources and gray literature that presents the business perspective. By going beyond academic, peer-reviewed sources, we are able to document real-world practices happening outside research contexts and examine differences by change initiator (investigator vs. restaurant-led strategies). Additionally, we also aim to expand knowledge beyond chain-based, corporate restaurants to include data regarding independently owned restaurants. This emphasis is important given the distinct differences in business approaches that exist between restaurants, which may impact how they approach the promotion of healthier eating.

## 2. Materials and Methods

We selected the following databases for searching the literature: MEDLINE (Medical Literature Analysis and Retrieval System Online), Cumulative Index of Nursing and Allied Health Literature (CINAHL), PsycINFO, and Business Source Complete. We created separate searches for each database to locate peer-reviewed and gray literature regarding health promotion and economic decision-making in restaurants, using combinations of controlled vocabulary such as medical subject headings (MeSH) terms and keywords to examine business and health-related records separately. This approach allowed us to capture business-focused sources, which may have been lost in a combined search. Food establishment related search terms included restaurants, fast foods, and take-aways; health-related terms included food habits, health promotion, and eating behavior; business-related search terms included commerce, marketing, and profits. Searches were limited, bounding the publication date range to January 2000–February 2020 (when the search was conducted) and English language. Books and theses were excluded. Additional records were culled from reference lists in review studies.

After duplicate records were removed, each record was independently assessed by two reviewers, using the Covidence systematic review software [18]. Records were included if the focus was on restaurants, describing restaurant-level, consumer-targeted strategies with a health-related aim. Sources were excluded if they were not restaurant-based (i.e., based on markets, institutions, or in laboratory settings), or if the paper did not entail a change strategy or intervention (i.e., mostly descriptive or epidemiological studies examining, for example, health outcomes from foods away from home or the characteristics of consumers eating foods away from home). We also excluded sources focused on street food vending, food trucks, bars, and cafes (if focusing only on coffee/beverages). To limit the scope of the review, we also excluded sources that focused on policy evaluation and review studies.

**Figure 1:**
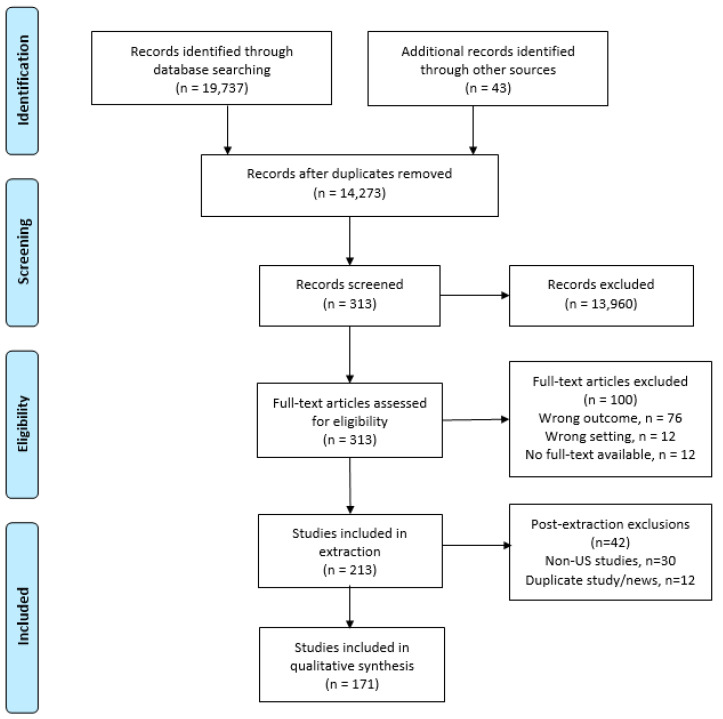
Flowchart for scoping review.

A total of 213 records were eligible for extraction. The extraction form collected information about the strategies addressed, barriers, motivation, and reported outcomes, as open fields, extracted directly from the source and then coded with codes developed a priori, based on insights gained via the screening process [19]. New codes were added as needed. The codes for strategies were developed based on previous research concerning healthful consumer environments in restaurants [20,21]. The extraction form was tested and revised with the research team before full implementation. Upon the completion of the extractions, each record was reviewed by a second reviewer for completion and accuracy, followed by a final check by the lead investigator. Conflicts encountered during the screening, extraction, and quality check were resolved during weekly team meetings. Prior to analysis, data cleaning procedures included revising codes and eliminating records that provided duplicate information, such as media articles discussing the same news or multiple intervention studies examining the same intervention. The records were compared, and the complete record was kept, adding information from the other record(s) as needed [22]. As a final step, we excluded sources that discussed strategies implemented outside of the United States (n = 30) to have a more uniform national context for the review. The screening and review process (Figure 1) resulted in a total of 171 records for analysis.

The analysis compared the main areas of interest (strategies, motivation, barriers, and outcomes) by two main factors: type of restaurant and strategy initiator (restaurant or investigator). The sources were coded as to whether they addressed independently owned restaurants, corporate/chain-based restaurants, or both. When restaurant type was not discernible from the source, the reviewers used an Internet search to locate the restaurant and determine the type.

## 3. Results

### 3.1. Sample Description

The review included a total of 171 sources. These included gray literature, such as restaurant news/reports (n = 109) [23,24,25,26,27,28,29,30,31,32,33,34,35,36,37,38,39,40,41,42,43,44,45,46,47,48,49,50,51,52,53,54,55,56,57,58,59,60,61,62,63,64,65,66,67,68,69,70,71,72,73,74,75,76,77,78,79,80,81,82,83,84,85,86,87,88,89,90,91,92,93,94,95,96,97,98,99,100,101,102,103,104,105,106], and health-related reports (n = 15) [107,108,109,110,111,112,113,114,115,116,117,118,119,120,121]; general news sources (n = 15) [122,123,124,125,126,127,128,129,130,131,132,133,134,135,136]; and peer-reviewed literature, including health and nutrition studies (n = 28) with a focus on restaurant intervention protocols and evaluations [137,138,139,140,141,142,143,144,145,146,147,148,149,150,151,152,153,154,155,156,157,158,159,160,161,162,163,164], and a few coming from other disciplines such as hospitality, marketing, and social sciences (n = 4) [165,166,167,168]. The majority of sources (n = 141) described strategies that were initiated by restaurants [23,24,25,26,27,28,29,30,31,32,33,34,35,36,37,38,39,40,41,42,43,44,45,46,47,48,49,50,51,52,53,54,55,56,57,58,59,60,61,62,63,64,65,66,68,69,70,71,72,73,74,75,76,77,78,79,80,81,82,83,84,85,86,87,88,89,90,91,92,93,94,95,96,97,98,99,100,101,102,103,104,105,106,107,108,109,110,112,113,114,115,116,117,118,119,120,121,122,123,124,125,126,127,128,129,130,131,132,133,134,135,136,144,145,164,168,169,170,171,172,173,174,175,176,177,178,179,180,181,182,183,184,185,186,187,188,189,190,191,192,193], in mostly gray literature and media sources, with three exceptions [145,164,168]. The rest (n = 30) described changes that were investigator-led, as part of nutrition-healthy eating focused intervention studies [111,137,138,139,140,141,142,143,144,146,147,148,149,150,151,152,153,154,155,156,157,158,159,160,161,162,163,165,166,167].

The scoping review used the record as the unit of analysis (as opposed to restaurants) because the information could not be easily extracted to each unique restaurant as multiple restaurants were included in most sources. Included sources addressed a total of 236 unique restaurants (188 unique corporate/chain-based restaurants and 48 independently owned restaurants). The number of restaurants evaluated in individual studies varied widely, from sources addressing a single restaurant or chain, to a maximum of 85, with an average of 6 restaurants per study. The majority of sources (n = 132) focused on corporate restaurants [23,24,25,26,27,28,29,30,31,32,33,34,35,36,37,38,39,40,41,42,44,48,49,50,51,52,53,54,55,57,58,59,61,62,63,64,65,66,67,68,70,71,72,73,74,75,76,77,78,79,80,81,82,83,84,85,87,88,89,90,91,92,94,95,96,97,98,99,100,101,103,104,105,106,107,108,109,110,112,113,114,115,116,117,118,119,120,122,123,124,125,126,127,128,129,130,131,132,134,135,136,139,143,153,154,156,160,166,169,170,171,172,173,174,175,176,177,178,179,180,181,182,183,184,186,187,188,189,190,191,192,193], mostly describing changes initiated by restaurants (n = 125). Fewer sources focused on independently owned restaurants (n = 27) [46,47,56,60,69,111,121,133,137,138,140,141,142,144,145,146,147,148,149,152,157,159,163,164,165,167,168], describing changes initiated by restaurants (n = 10) and investigators (n = 17). A few sources addressed both types of restaurants (n = 12) [43,45,86,93,102,150,151,155,158,161,162,185], with an even split by initiator. While most sources presented strategies that were implemented, a few (n = 19) described restaurant-initiated planned changes or pledges in corporate restaurants [34,53,74,77,80,82,90,98,100,104,108,109,122,124,131,135,171,178,179]. We did not exclude these sources, as they provided information regarding the motivations and barriers associated with these plans.

### 3.2. Consumer-Facing Strategies for Healthier Eating

More than half of the sources (57%) reported the provision of healthier food options, including offerings lower in calories, fats, and sugar, as well as mentions of healthier offerings in general [25,29,31,32,35,36,39,40,41,42,43,44,45,46,47,48,51,55,57,58,59,60,62,66,67,68,69,70,73,74,75,76,78,79,80,81,83,84,85,86,88,90,91,93,94,95,96,97,98,101,102,105,106,108,109,112,113,114,117,118,120,121,122,123,124,125,131,132,135,139,140,141,143,145,148,150,151,153,156,159,162,163,164,165,169,171,173,174,175,176,178,181,182,185,187,188,189,190,193]. Fewer sources reported on specific healthful food offerings, such as increased availability of fruits, vegetables, salads, whole grains, and healthier beverages (Table 1). Almost one-quarter of sources (22%) reported on environmentally sustainable or “clean” offerings (i.e., Hormone-free meat, no high-fructose corn syrup), including using organic ingredients, local foods, or foods that were prepared without additives or artificial ingredients [23,35,45,48,56,66,67,69,72,75,76,80,86,89,91,92,93,94,99,100,102,103,104,106,110,115,119,120,121,132,133,134,135,136,168,172,175]. A small percentage of sources (11%) discussed restaurant offerings that responded to trends, notably “gluten-free” and “low carb” options [24,26,28,29,31,33,62,85,92,93,99,105,106,116,133,145,179,185,187]. These two strategies were only found in sources describing restaurant-initiated changes (Table 1).

Aside from changes in food offerings, strategies also encompassed environmental facilitators for healthy choices. The second most frequently reported strategy was the promotion of healthier options (30%) [24,29,31,34,40,41,52,54,56,59,60,66,70,79,80,81,87,89,91,116,117,120,121,131,137,138,139,141,143,147,148,149,150,151,154,155,158,159,160,161,165,168,171,172,173,179,180,183,185,186,187,189], including through menu highlights of healthier choices or, table tents or posters, but also included conducting nutrition education in the restaurant (n = 2) [56,186] and having waitstaff trained in the promotion of healthier options (n = 3) [101,159,162]. The second most common environmental strategy was the provision of nutrition information (e.g., Menu nutrition labeling) (25%) [24,28,29,30,31,32,34,36,53,66,70,77,81,82,110,111,114,116,121,126,131,137,139,141,142,144,146,147,154,160,161,162,165,167,169,170,172,181,182,183,185,186,192], followed by the provision of smaller portions, either by decreasing portion sizes and/or offering a smaller portion option to customers (17%) [31,39,44,49,51,55,61,70,73,74,76,83,88,93,112,114,125,129,140,146,150,152,155,157,159,162,180,184,192], promoting healthy substitutions (e.g., side salad instead of fries) (12%) [31,32,39,41,53,71,73,79,83,87,91,93,107,124,130,136,154,166,171,190], and pricing schemes to promote healthier options (e.g., healthy combination meals) (11%) [29,54,59,66,79,91,114,137,139,141,147,153,154,158,159,161,167,189,191]. Overall, environmental facilitators were more commonly found in sources focused on independently owned restaurants and/or describing investigator-led changes (Table 1). Aside from these healthy eating facilitating strategies, a few sources (5%) described the promotion of physical activity (e.g., partnering with athletes to promote exercise) [29,34,40,80,81,126,179,180,191]. This was only found in corporate restaurant, such as major translational chains, as a restaurant-initiated strategy.

Fifteen records presented restaurants with a healthy business model at the time of opening [32,57,60,63,68,69,75,121,133,134,168,172,176,181,193]—a restaurant-led strategy. Most addressed corporate/chain-based restaurants with a healthful focus (n = 10) [32,57,63,68,75,134,172,176,181,193], while only five were classified as independently owned [60,69,121,133,168]. Following the overall sample trend, the most common strategy was the provision of healthier offerings in general (n = 7) [32,57,68,75,176,181,193], followed by restaurants that offered more vegetables-based dishes (n = 6) [63,75,121,133,172,193]. Compared with the overall sample, environmental strategies were not as salient. Only a few sources discussing healthy restaurants business models described promotion of healthy offerings (n = 4) [60,121,168,172] or nutrition information provision (n = 4) [32,121,172,181], the second and third most common strategies in the larger sample, respectively (Table 1).

### 3.3. Reported Motivations and Barriers for Strategy Implementation

The reporting of motivations and barriers for strategy implementation was not consistent across sources. Motivation was not discernable in 23% of the sample [38,114,115,117,118,119,128,129,137,138,146,147,149,152,156,160,162,163,165,166,167], although it was high among investigator-initiated changes (70%) [111,137,138,139,146,147,149,150,151,152,153,154,156,157,158,160,162,163,165,166,167]. Among those sources where motivation was discernible, these were customer centered or profit-driven, including the most common motivation, perceived customer demand (56%) [23,24,26,27,28,29,31,33,36,39,40,42,44,45,48,50,51,52,55,57,58,64,65,66,67,68,69,70,72,75,76,77,79,83,85,86,89,91,92,93,95,96,97,98,99,100,101,102,103,104,105,106,116,121,123,132,134,135,136,144,164,168,169,174,175,176,178,181,182,183,184,185,187,193] (Table 2).

The other motivating forces were grouped as following: personal factors, top-down pressure, or external, mostly related to food sourcing (2%) [88,94]. The second most common motivation was the desire to improve community health (27%) [23,35,43,46,47,55,56,60,66,68,74,75,82,87,90,94,95,104,110,121,126,133,141,142,145,148,159,161,164,168,183,184,189,191,192], followed by responding to public health criticism (22%) [25,27,30,34,37,44,45,49,66,67,71,84,112,120,122,124,127,131,132,134,140,170,173,174,177,180,186,188,190] (Table 2).

The analysis revealed differences by type of restaurant and initiator. Among sources focused on independently owned restaurants, the most prevalent motivation was a desire to improve community health (81%) [46,47,56,60,121,133,141,142,145,148,159,164,168], followed by perceived customer demand (60%) [69,121,144,164,168]. In sources focused on corporate restaurants, the second most common motivation was public health criticism, or engaging in healthy eating promoting strategies to counteract views of corporate (fast food) establishments as unhealthy (25%) [25,27,30,34,37,44,49,66,67,71,84,112,120,122,124,127,131,132,134,170,173,174,177,180,186,188,190]. When changes were initiated by restaurants, meeting perceived customer demand was the main motivation (60%) [23,24,26,27,28,29,31,33,36,39,40,42,44,45,48,50,51,52,55,57,58,64,65,66,67,68,69,70,72,75,76,77,79,83,85,86,89,91,92,93,95,96,97,98,99,100,101,102,103,104,105,106,116,121,123,132,134,135,136,164,168,169,174,175,176,178,181,182,183,184,185,187,193], followed by wanting to improve community health (25%) [23,35,43,46,47,55,56,60,66,68,74,75,82,87,90,94,95,104,110,121,126,133,145,164,168,183,184,189,191,192] and addressing public health criticism (23%) [25,27,30,34,37,44,45,49,66,67,71,84,112,120,122,124,127,131,132,134,170,173,174,177,180,186,188,190] (Table 2). In sources addressing investigator-led changes, the motivations also included a desire to improve community health (56%) [141,142,148,159,161], followed by seeing the participation in the intervention as an opportunity to promote the business (44%) [140,143,144,155], as a common incentive provided for restaurants to participate in health promoting interventions.

When examining the subset of records presenting healthy restaurant businesses models, these were mostly motivated by seeing health as a business opportunity (n = 11) [57,60,63,68,69,75,133,168,172,181,193], followed by perceived customer demand (n = 10) [57,68,69,75,121,134,168,176,181,193], and wanting to improve community health (n = 6) [60,68,75,121,133,168].

Close to three-fourths of the sources (70%) did not discuss barriers for the implementation of health promoting strategies [23,24,25,26,27,28,30,32,33,34,35,36,38,39,40,41,43,45,46,50,52,53,54,56,57,58,60,61,62,63,68,70,71,72,74,75,77,78,79,80,81,82,83,84,86,87,89,91,93,94,95,96,97,98,99,100,102,103,104,105,106,107,108,109,110,111,112,114,115,116,117,118,121,122,123,125,126,127,128,132,133,135,136,138,139,142,146,148,152,153,154,156,157,160,162,165,166,167,169,170,171,172,173,174,177,178,179,180,181,182,184,185,186,187,188,189,191,192,193], an issue prevalent in sources describing changes initiated by restaurants (73%) [23,24,25,26,27,28,30,32,33,34,35,36,38,39,40,41,43,45,46,50,52,53,54,56,57,58,60,61,62,63,68,70,71,72,74,75,77,78,79,80,81,82,83,84,86,87,89,91,93,94,95,96,97,98,99,100,102,103,104,105,106,107,108,109,110,112,114,115,116,117,118,121,122,123,125,126,127,128,132,133,135,136,169,170,171,172,173,174,177,178,179,180,181,182,184,185,186,187,188,189,191,192,193], and describing changes in corporate restaurants (74%) [23,24,25,26,27,28,30,32,33,34,35,36,38,39,40,41,50,52,53,54,57,58,61,62,63,68,70,71,72,74,75,77,78,79,80,81,82,83,84,87,89,91,94,95,96,97,98,99,100,103,104,105,106,107,108,109,110,112,114,115,116,117,118,122,123,125,126,127,128,132,135,136,139,153,154,156,160,166,169,170,171,172,173,174,177,178,179,180,181,182,184,186,187,188,189,191,192,193] (Table 3).

When examining barriers, these included issues faced when implementing the strategy, and also reported drawbacks, including from lessons learned when recruiting restaurants for public health interventions as well as reasons cited by reports for restaurants nor engaging in health promoting strategies. The most commonly found barriers were those related to revenue, including concern about revenue decrease (42%) [31,37,44,49,69,90,101,120,124,137,140,144,147,149,151,155,158,161,164,175,183,190] and concern regarding customer acceptance (48%) [29,42,47,48,49,51,55,59,64,66,67,73,76,88,90,92,113,120,124,129,134,158,164,168,176]. The third most common barrier related to food costs and sourcing (23%) [29,44,48,55,67,85,119,124,131,150,159,176] (Table 3). While reported in lower numbers, Table 3 presents a variety of important logistical barriers, aside from food sourcing. These included issues related to time constraint (21%) [65,67,124,140,144,145,151,155,159,161,164], limitations regarding recipes and food preparation (8%) [65,67,144,163], kitchen or restaurant space constraints (6%), and staff constraints (e.g., Culinary training and turnover) (4%) [158,164] (Table 3).

Profit-related barriers were salient across all records when compared by initiator and restaurant type. Personal barriers were more prevalent in sources examining independently owned restaurants and those initiated by investigators. On the other hand, food sourcing constraints were more common in sources addressing corporate restaurants (29%) [29,44,48,55,67,85,119,124,131,176] and initiated by restaurants (26%) [29,44,48,55,67,85,119,124,131,176] (Table 3).

Among sources presenting healthy restaurant business models, very few reported barriers, including worry about customer acceptance (n = 3) [134,168,176], worry about revenue decrease (n = 1) [69] and issues regarding food costs/sourcing (n = 1) [176].

### 3.4. Reported Outcomes

Only 36% (n = 64) of the sources reported outcomes associated with the strategies [24,27,29,31,36,39,46,55,56,60,62,65,68,73,75,84,88,90,94,103,112,113,121,131,134,137,138,139,140,141,142,143,144,145,146,147,148,149,150,151,152,153,154,155,156,157,158,159,160,161,162,163,165,166,167,172,176,177,180,182,185,189]. Reporting was most common in investigator-led strategies, where only one record failed to report outcomes [111]. Overall, the most common outcomes were classified as revenue positive, specifically, customer acceptance of strategy or change (27% of reporting sources) [46,55,60,84,88,90,142,143,145,146,150,152,156,158,161,165,177] and increase in revenue (26% of reporting sources) [27,31,56,65,68,75,121,131,134,147,148,153,158,177,185,189] (Table 4). Sources presenting investigator-led changes mostly reported health-related outcomes, notably improvements in eating behaviors (48%) [137,138,139,140,142,147,149,150,153,154,157,159,163,165] and outcomes associated with the successful intervention implementation, including improvements to the restaurant consumer nutrition environment (34%) [140,141,144,147,148,151,155,158,162,165] and acceptance of the intervention by restaurants (21%) [141,144,147,148,161,165] and customers (31%) [142,143,146,150,152,156,158,161,165].

When examining the records describing healthy restaurant businesses models, seven of the 15 sources had potential insights on outcomes [60,68,75,121,134,172,176]. Most were positive, including increased revenue (n = 4) [68,75,121,134], restaurant gaining visibility (n = 1) [172], and customer acceptance (n = 1) [60]. Only one source included revenue decrease and lack of customer acceptance [176].

## 4. Discussion

The goal of this scoping review was to examine the engagement of a wide range of restaurants in strategies or innovations to facilitate healthier eating, calling attention to independent as well as corporate or chain restaurants. Expanding beyond investigator-initiated research sources, and specifically with the inclusion of media and gray literature sources, this review provides insights from the perspective of the restaurant—a research gap in the existing academic literature as noted in pasts reviews [17]. Our assessment of real-world practices suggests that restaurants are responding to the increased customer demand for healthier offerings [194] and that this response varies according to restaurant type and who initiates the change.

Changes initiated by restaurants, mostly in corporate-owned restaurants, sought to increase the availability of healthier offerings, in general. Investigator-initiated changes and those in independently owned restaurants were mostly environmental in nature, notably the promotion of available healthier choices. This may be due to these changes being driven mostly by investigators seeking to work with healthful offerings already offered (rather than telling restaurants what foods to offer). While our review does not provide confirmation on the rationale behind this difference, this may be potentially due to investigators seeking to lower the risk of participation for the restaurants or the tangible and intangible costs involved in creating new dishes. This trend concurs with past reviews focusing on community-based restaurants, where the point of sale promotion and provision of information was the more salient intervention, compared with the increase in healthier offerings [8]. Compared with independently-owned restaurants, corporate restaurants have more resources to develop and test new offerings, respond to perceived consumer trends or avoid alienating the healthy eater in the party [195]. Independently owned restaurants often cater to a smaller customer base and operate under much narrower profit margins that can limit the experimentation with new offerings unless it is driven by an intrinsic motivation to innovate. Future research can best be used to explore these differences, examining the innovation processes driving innovation in restaurants, examining in greater detail these processes while accounting for restaurant type, such as corporate vs. independently owned, as well as categorizing establishments by price-point and target clientele. This information will provide public health practitioners more information from the business perspective, highlighting leverage points when seeking to collaborate with restaurants in future interventions.

While the changes in offerings may be encouraging, less reporting was found when examining the increase of specific health-promoting foods, as in the case of fruits, vegetables, whole grains, and salads. The innovations or changes in regards to offerings demonstrated the restaurant response to food trends, including changes made in concordance to diet “fads”, as in the case of low carb or high protein offerings, which respond to popular diets as in the case of the Atkins diet or Keto diets, among others [196], and in concordance with concerns over environmental sustainability and additives in the food supply, where restaurants were seeking to promote plant-based diets, as well as dishes made with local foods or additive-free (“clean”) offerings [197,198].

The analysis revealed four factors that were found to influence restaurants’ ability to engage in healthy eating promotion strategies: perceptions regarding customer demand, expectations concerning revenue, views concerning community health, and food sourcing. Perceptions concerning demand and profitability were salient as both motivation and barrier, demonstrating the potentially subjective and context-dependent nature of this important factor. Glanz et al. conducted a study on menu planning where they documented the reluctance to increase healthier offerings if consumer demand is low, as these offerings can also risk spoilage and may need additional labor in preparation [195]. The sourcing of fresh ingredients, as in the case of fruits and vegetables, can also be inconsistent, further increasing the risk for these healthier offerings, which may not be viewed as profitable in the first place. At the same time, our findings concerning reported outcomes reveal that the engagement in health-promoting strategies can be good for business, given the reported increase in profit, consumer acceptance, and business visibility. While our examination of outcomes was not systematic and limited to what was reported in the source, this finding concurs with Blake et al. scoping review of the effect of health-promoting strategies on business outcomes, where 45% of sources reported favorable business outcomes and only 15% were unfavorable (34% classified as “neutral”). These included retailer satisfaction, perceived customer satisfaction, and those influencing profit (i.e., Amount spent by transaction) [17]. Public health research articles focused mostly on consumer-related outcomes, including acceptability and purchasing behaviors. In concordance with Blake et al., outcomes were not well reported in terms of revenue or profit, especially in public health intervention studies [17]. These were mostly assessed relying on restaurant-provided sales data, presenting logistical barriers for researchers to obtain.

Aside from business outcomes, our findings revealed the importance placed on community health—a factor that may also be subjective and highly personal. While restaurants may be nudged to engage in healthy eating promotion strategies, by policy mandate or an investigator-led effort, the success and sustainability of such strategies will depend on the value restaurant owners place on health and whether owners perceive restaurants have a role in improving health in their communities. Lack of interest was an important barrier in sources describing investigator-led strategies, underscoring the importance of ownership and engagement when working with restaurant stakeholders. Creating the change needed cannot be sustainably done by public health interventionist plea or imposition.

Our examination of sources describing healthy restaurant business models reveals potential case studies for future research where we see a confluence of key motivations, resulting in healthy restaurant environments. These restaurants are the result of a recognition of healthful offerings as profitable, meeting perceived market demand, while also being driven to improve community health. Our examination of these restaurants was facilitated by the inclusion of gray literature, as these were not featured as part of academic intervention studies. Moving forward, building on this approach, future research can continue to examine healthy restaurant business models, to gather potential lessons learned for other restaurants wishing to undertake changes to follow, and to document these as success stories that can help model desired behaviors for other restaurants in the future.

### Strengths and Limitations

This review used a scoping methodology, expanding the review to include new sources of information seldom incorporated in systematic reviews of interventions. This allowed us to examine changes restaurants have undertaken, independent of a public health intervention, as a business decision. The results show the trend in the industry to cater to perceived growing customer demand for healthier options in restaurants and potential changes in social norms concerning restaurants being viewed primarily as a site for indulgence. At the same time, our study is limited by publication bias and lack of reporting on key aspects, including outcomes, barriers, and motivation. We also cannot link specific strategies to their corresponding barriers, motivations, and outcomes. Our results are also limited to the US context. Our exclusion of non-US sources was primarily driven to provide a homogeneous context to our analysis, but our search and screening process yielded a relatively low number of English records, to begin with (n = 30), showcasing the need for more research in other context and reviews examining sources in languages other than English. Lastly, the sources included in this review were compiled across two decades preceding the onset of COVID-19. While this allowed us to examine changes implemented before the pandemic, the restaurant industry has been changing, responding to the pandemic and a climate of uncertainty, underscoring the need to further research how these adaptations may influence the implementation of future healthy eating promoting strategies.

## 5. Conclusions

This scoping review contributes to food environment research by going beyond more commonplace disciplinary boundaries used in other review studies focused on public health research articles. Our review demonstrated that most of the evidence to date was found in the real world and not in academic, peer-reviewed sources. We were able to bring forth practice-based evidence, showcasing the business perspective, as an important consideration when seeking to change food environments. This approach can be considered in future work seeking to understand system dynamics shaping food environments and persisting diet-related disparities, pushing researchers to engage in interdisciplinary and intersectoral collaborations to assess and learn from real-world practices. Our approach also allowed us to examine key differences by restaurant type, underscoring the importance of tailored approaches when seeking to address independently owned versus corporate restaurants. The former is in need of incentives-based approaches, such as subsidies or tax incentives, to create healthful changes.

The engagement of restaurants in healthy eating promoting strategies and innovations demonstrates the growing recognition of demand and need for these innovations [194]. This is further evidenced by restaurants designed with healthy eating in mind, as found in the subsample of records regarding these restaurants [32,57,60,63,68,69,75,121,133,134,168,172,176,181,193]. At the same time, these strategies, particularly those involving changes in offerings, do not seem as widely adopted in independently owned restaurants compared to corporate ones. More work is needed to create incentives that facilitate such changes, potentially including policy interventions that support these restaurants, while recognizing their important role in local communities and needs as businesses. The COVID-19 pandemic has highlighted the fragility of the restaurant industry [199], but also the adaptability of the sector when faced with the need to innovate. More research is needed to examine whether the innovations examined in this study will continue to cater to healthy eaters, or if these efforts will be halted given business-related concerns, issues with the food supplies or the uncertainty of regulations targeting the industry, in response to the pandemic.

## Figures and Tables

**Table 1:** Consumer-facing strategies reported, overall and by type of restaurant addressed in source and initiator (n = 171).

Strategies	Overall (n = 171)		Type of Restaurant(s) Addressed in Source						Initiator of Change			
			Corporate-Owned (n = 132)		Independently Owned (n = 27)		Both (n = 12)		Restaurant-Initiated (n = 141)		Investigator-Initiated (n = 30)	
	n	%	n	%	n	%	n	%	n	%	n	%
Changes in food availability												
General increase in healthy offerings	98	57%	77	58%	13	48%	8	67%	86	61%	12	40%
Fruit	29	17%	26	20%	2	7%	1	8%	27	19%	2	7%
Salads	24	14%	23	17%	1	4%	0	0%	24	17%	0	0%
Vegetables	29	17%	19	14%	5	19%	5	42%	27	19%	2	7%
Whole grains	12	7%	11	8%	1	4%	0	0%	11	8%	1	3%
Healthier beverages	15	9%	9	7%	2	7%	4	33%	9	6%	6	20%
Sustainable/clean offerings	38	22%	29	22%	5	19%	4	33%	38	27%	0	0%
Diet “fad” offerings	19	11%	15	11%	2	7%	2	17%	19	13%	0	0%
Environmental facilitators												
Provision of nutrition information	43	25%	30	23%	10	37%	3	25%	29	21%	14	47%
Changes in portion size offerings	29	17%	20	15%	5	19%	4	33%	21	15%	8	27%
Pricing schemes to favor healthy options	19	11%	12	9%	5	19%	2	17%	9	6%	10	33%
Promotion of healthy options	52	30%	34	26%	12	44%	6	50%	35	25%	17	57%
Encouraging healthy substitutions	20	12%	19	14%	0	0%	1	8%	18	13%	2	7%

**Table 2:** Reported motivations for engaging in healthy eating promotion strategies, overall and stratified by restaurant type addressed and change initiator.

Motivation			Type of Restaurant(s) Addressed in Source						Initiator of Change			
	Overall (n = 171)		Corporate-Owned (n = 132)		Independently Owned (n = 27)		Both (n = 12)		Restaurant-Initiated (n = 141)		Investigator-Initiated (n = 30)	
Motivation not reported	40	23%	25	19%	11	41%	4	33%	19	13%	21	70%
Profit-driven												
Perceived customer demand	74	56% ^1^	64	60%	5	31%	5	63%	73	60%	1	11%
Want to increase profit	9	7%	8	7%	1	6%	0	0%	8	7%	1	11%
Recognizing health as business opportunity	12	9%	8	7%	4	25%	0	0%	12	10%	0	0%
Following food trends	4	3%	3	3%	0	0%	1	13%	4	3%	0	0%
Business promotion opportunity	9	7%	6	6%	2	13%	1	13%	5	4%	4	44%
Intrinsic motivations/factors												
Want to improve community health	35	27%	20	19%	13	81%	2	25%	30	25%	5	56%
Desire to innovate	3	2%	1	1%	0	0%	2	25%	3	2%	0	0%
Top-down pressure												
Public health criticism	29	22%	27	25%	1	6%	1	13%	28	23%	1	11%
Mandates/guidelines	5	4%	4	4%	1	6%	0	0%	4	3%	1	11%
External factors												
Food sourcing/availability	2	2%	2	2%	0	0%	0	0%	2	2%	0	0%

^1^ percentages from this row forward were calculated using the number of records where motivation was discernible as denominator.

**Table 3:** Reported barriers for engaging in healthy eating promotion strategies, overall and stratified by restaurant type addressed and change initiator.

Barriers	Overall (n = 171)		Type of Restaurant(s) Addressed in Source						Initiator of Change			
			Corporate-Owned (n = 132)		Independently Owned (n = 27)		Both (n = 12)		Restaurant-Initiated (n = 141)		Investigator-Initiated (n = 30)	
Barrier not reported	119	70%	98	74%	14	52%	7	58%	103	73%	16	53%
Profit-related												
Worry about revenue decrease	22	42% ^1^	11	32%	7	54%	4	80%	13	34%	9	64%
Worry about customer acceptance	25	48%	21	62%	3	23%	1	20%	24	63%	1	7%
Customer demand for unhealthy options	3	6%	2	6%	1	8%		0%	2	5%	1	7%
Personal/intrinsic barriers												
Lack of interest or recognition regarding the role of restaurants in health promotion	9	17%	1	3%	5	38%	3	60%	2	5%	7	50%
Time constraints	11	21%	3	9%	5	38%	3	60%	5	13%	6	43%
Physical environment barriers												
Food sourcing constraints (cost, access)	12	23%	10	29%	1	8%	1	20%	10	26%	2	14%
Spatial limitations (kitchen or restaurant space)	3	6%	1	3%	2	15%		0%	1	3%	2	14%
Staff-related constraints issues (i.e., Staff turnover, knowledge and culinary skills.)	2	4%		0%	1	8%	1	20%	1	3%	1	7%
Food/recipe limitations	4	8%	2	6%	2	15%		0%	2	5%	2	14%

^1^ percentages calculated using the number of records where motivation was discernible as denominator.

**Table 4:** Reported outcomes, overall and stratified by restaurant type, addressed and change initiator.

Outcomes	Overall (n = 171)		Type of Restaurant(s) Addressed in Source						Initiator of Change			
			Corporate-Owned (n = 132)		Independently Owned (n = 27)		Both (n = 12)		Restaurant-Initiated (n = 141)		Investigator-Initiated (n = 30)	
	n	%	n	%	n	%	n	%	n	%	n	%
Outcomes not reported	109	64%	98	74%	6	22%	5	42%	108	77%	1	3%
Revenue positive												
Revenue increased	16	26% ^1^	10	29%	4	19%	2	29%	12	36%	4	14%
Customer accepted change	17	27%	7	21%	7	33%	3	43%	8	24%	9	31%
Restaurant promotion/visibility	6	10%	5	15%	0	0%	1	14%	4	12%	2	7%
Revenue negative												
Revenue decreased	2	3%	1	3%	1	5%	0	0%	1	3%	1	3%
Customer did not like change	2	3%	2	6%	0	0%	0	0%	2	6%	0	0%
Revenue neutral												
Mixed (customer acceptance)	2	3%	2	6%	0	0%	0	0%	2	6%	0	0%
Revenue not affected	2	3%	1	3%	1	5%	0	0%	0	0%	2	7%
Health-improving outcomes												
Customer improved eating behaviors	15	24%	4	12%	10	48%	1	14%	1	3%	14	48%
Restaurant accepted intervention (investigator-initiated only)	6	10%	0	0%	5	24%	1	14%		0%	6	21%
Restaurant nutrition environment improved	15	24%	5	15%	6	29%	4	57%	5	15%	10	34%
Restaurant staff increased nutrition knowledge	4	6%	0	0%	3	14%	1	14%	0	0%	4	14%
Other												
Mixed (restaurant acceptability)	1	2%	1	3%	0	0%	0	0%	1	3%	0	0%
No change in eating behaviors	2	3%	2	6%	0	0%	0	0%	0	0%	2	7%

^1^ percentages calculated using the number of records reporting outcomes.

## Data Availability

Not applicable.
